# Teeth Restored with Bulk–Fill Composites and Conventional Resin Composites; Investigation of Stress Distribution and Fracture Lifespan on Enamel, Dentin, and Restorative Materials via Three-Dimensional Finite Element Analysis

**DOI:** 10.3390/polym15071637

**Published:** 2023-03-25

**Authors:** Hakan Yasin Gönder, Reza Mohammadi, Abdulkadir Harmankaya, İbrahim Burak Yüksel, Yasemin Derya Fidancıoğlu, Said Karabekiroğlu

**Affiliations:** 1 Department of Restorative Dentistry, Faculty of Dentistry, Necmettin Erbakan University, Baglarbasi Street No: 4 Meram/Konya, Konya 42090, Turkey; 2 Department of Mechanical Engineering, University of Zanjan, Zanjan 45371-38791, Iran; 3 Faculty of Dentistry, Necmettin Erbakan University, Baglarbasi Street No: 4 Meram Konya, Konya 42090, Turkey; 4 Department of Oral Diagnose and Radiology, Faculty of Dentistry, Necmettin Erbakan University, Baglarbasi Street No: 4 Meram Konya, Konya 42090, Turkey; 5 Department of Pediatric Dentistry, Faculty of Dentistry, Necmettin Erbakan University, Konya 42090, Turkey; 6 Department of Restorative Dentistry, Faculty of Dentistry, Necmettin Erbakan University, Konya 42090, Turkey

**Keywords:** finite element analysis, bulk–fill composite, resin composite, stress distribution, fatigue

## Abstract

Objectives: the aim of this study was to examine the stress distribution of enamel, dentin, and restorative materials in sound first molar teeth with restored cavities with conventional resin composites and bulk–fill composites, as well as to determine their fracture lifetimes by using the three-dimensional finite element stress analysis method. Materials and Methods: an extracted sound number 26 tooth was scanned with a dental tomography device and recorded. Images were obtained as dicom files, and these files were transferred to the Mimics 12.00 program. In this program, different masks were created for each tooth tissue, and the density thresholds were adjusted manually to create a three-dimensional image of the tooth, and these were converted to a STL file. The obtained STL files were transferred to the Geomagic Design X program, and some necessary adjustments, such as smoothing, were made, and STP files were created. Cavity preparation and adhesive material layers were created by transferring STP files to the Solidworks program. Finally, a FE model was created in the ABAQUS program, and stress distributions were analyzed. Results: when the bulk–fill composite and conventional resin composite materials were used in the restoration of the cavity, the structures that were exposed to the most stress as a result of occlusal forces on the tooth were enamel, dentin, restorative material, and adhesive material. When the bulk–fill composite material was used in restoration, while the restorative material had the longest fracture life as a result of stresses, the enamel tissue had the shortest fracture life. When the conventional resin composite material was used as the restorative material, it had the longest fracture life, followed by dentin and enamel. Conclusion: when the bulk–fill composite material was used instead of the conventional resin composite material in the cavity, the stress values on enamel, dentin, and adhesive material increased as a result of occlusal forces, while the amount of stress on the restorative material decreased. In the fracture analysis, when the bulk–fill composite material was used instead of the conventional resin composite material, a decrease in the number of cycles required for the fracture of enamel, dentin, and restorative materials was observed as a result of the forces generated in the oral cavity.

## 1. Introduction

Dental caries represent one of the most common and preventable diseases. People are susceptible to cavities throughout their lives, and cavities can result in toothache or tooth loss if left untreated. Caries are among the most important causes of pain in the mouth and tooth loss [1,2,3,4,5]. They can be prevented in the early stages, but if proper care and treatment are not adhered to, they will continue to develop and rapidly destroy the tooth tissue [1]. Restorative materials to be applied to cavities must be carefully selected. The selection of appropriate restorative materials leads to reductions in biofilm layers, reductions in the formation of caries, and the risk of periodontal disease, as well as less stress accumulation in dental tissues [6]. Improper stress distribution and biofilm layers can cause a restoration to detach from the cavity, as well as leakage problems in the restoration and retention failure [7,8]. Resin-containing composites were mainly developed to restore the aesthetics and function of teeth in their natural state, but they are also widely used in class I and II restorations in dentistry today [9,10,11]. Mechanical properties, such as hardness, durability, and high wear resistance, are sought in resin composites to be used in the posterior region [12]. The bulk–fill composite material, which is used as another restorative material, can be polymerized in one go when used up to 4–5 mm thickness. Thus, as the layering technique that needs to be applied in composite resins is eliminated, the restoration time is even shorter [13]. The bulk insertion technique often needs to be used on posterior teeth, which are exposed to more stress. Therefore, bulk–fill composites should have adequate mechanical properties [14]. In dentistry, the ability to resist chewing forces in the oral cavity is one of the most important requirements for dental restorative materials. The elastic modulus plays a very important role in the longevity of the restoration and the strength of the surrounding tooth tissue. Ideally, the elastic properties of restorative materials should be close to those of dental tissue to provide a more uniform stress distribution. However, teeth consist of enamel and dentin tissue, which are elastically different from each other. For this, two different restorative materials should be used, and one of them should be chosen as a standard [15,16,17,18]. The properties of restorative materials affect the stress distribution in dental tissues and thus the durability of the restoration. A lower stress concentration in the tooth and restoration is a good indicator for restorative materials [19,20,21]. The elastic module has a very important effect on the longevity of the restoration and the preservation of the strength of the tooth tissue. Ideally, the elastic properties of restorative materials should be similar to those of the tooth structure to provide a more uniform stress distribution. The finite element method (FEM) is recognized in the literature as a widely used and reliable tool for simulation and computational analyses [15,16,17,18].

Finite element analysis (FEM) is frequently used in dental applications, especially for strength analysis in the restorative field and for the evaluation of different restorative materials [22]. While it is not possible to repeat an experiment many times in clinical studies in dentistry, experiments that utilize this method can be easily repeated using computer programs [23,24,25,26]. This analysis method provides researchers with the opportunity to perform static and dynamic analyses under various variables in a non-invasive way [27,28]. Compared to laboratory tests, the finite element stress analysis method has many advantages, such as not needing living tissues, providing the ability to manipulate variables, and being less time-consuming than many other methods [24,29]. In addition to all of these features, this analysis method has some limitations, e.g., it presents difficulties in transferring biological structures to the computer environment, has high rates of human-induced errors, as well as long computation times, due to the complexity of the numerical calculations [19,29].

The aim of the study was to examine the stress distribution and fracture life of enamel, dentin, and restorative materials in first molar teeth in which cavities were restored with resin-containing composites and bulk–fill composites, which are frequently used in dentistry, using the three-dimensional finite element stress analysis method.

The null hypothesis of the study was that the restorative materials do not affect the stress distribution transmitted to the dental tissues, and the tooth tissues have no effect on the fracture life of the restorations.

## 2. Materials and Methods

**Figure 1:**
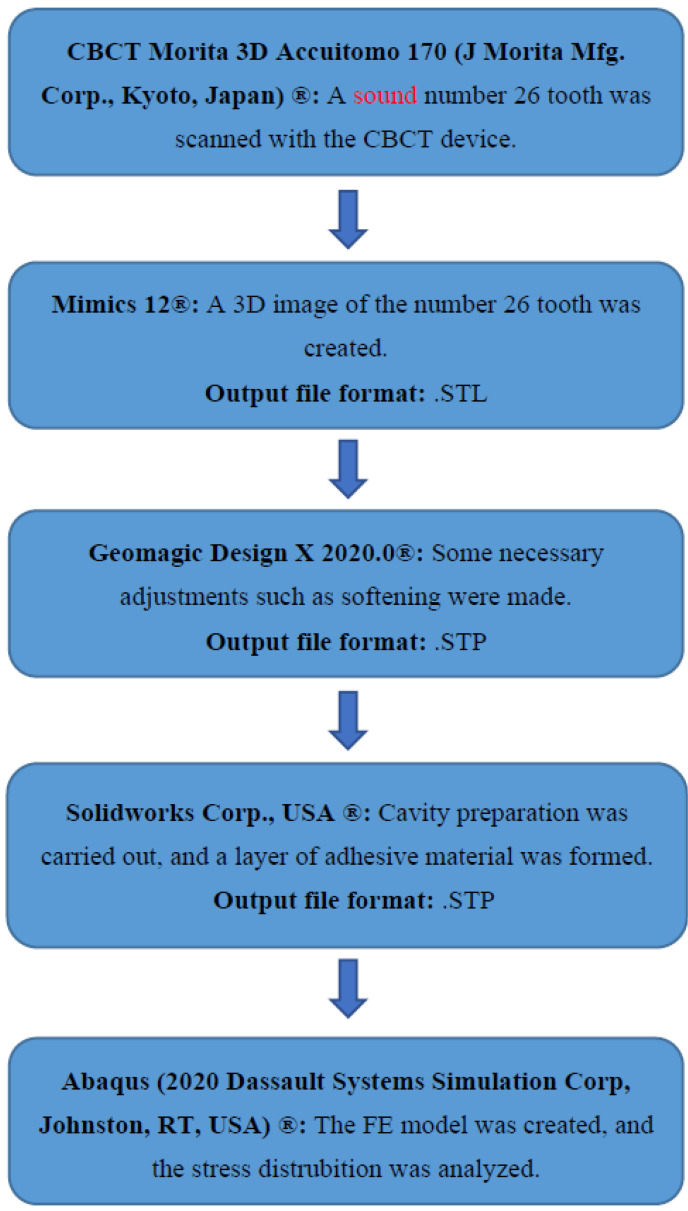
Sequential set of equipment and software used for finite element stress analysis.

The sequential set of equipment and software used for the finite element stress analysis of maxillary first molars restored with composite resin and bulk–fill composite materials is shown in Figure 1.

**Figure 2:**
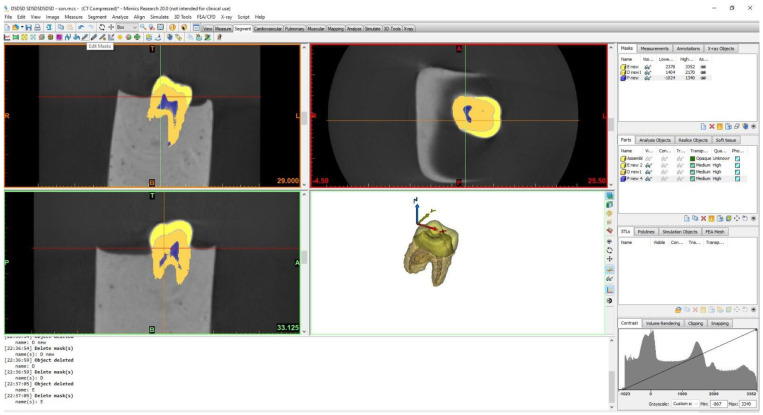
The three−dimensional model of the sound tooth was created manually in the Mimics program.

The three-dimensional geometry of a sound number 26 tooth, which was captured using a dental tomography (DA1) device, was scanned. Cone beam computerized tomography (CBCT) images were taken using Morita 3D Accuitomo 170 (J Morita Mfg. Corp., Kyoto, Japan). The size of the imaging volume was a cylinder with a diameter of 40 × height of 40 mm at the X-ray rotational center. The images were taken under the exposure condition of 90 kVp (X-ray tube voltage) and 5 mA (value of the electric current), which were the standard parameters and could be changed for different subjects. The images were taken using 160 qm and 17.5-second exposure time parameters. The images were acquired as dicom files. These obtained files were then transferred to the Materialize Interactive Medical Image Control System (Mimics 12.00, Leuven, Belgium) program, different masks were created for each tooth tissue (enamel, dentin and pulp), and density thresholds were manually adjusted to create the correct anatomy of the tooth (Figure 2).

**Figure 3:**
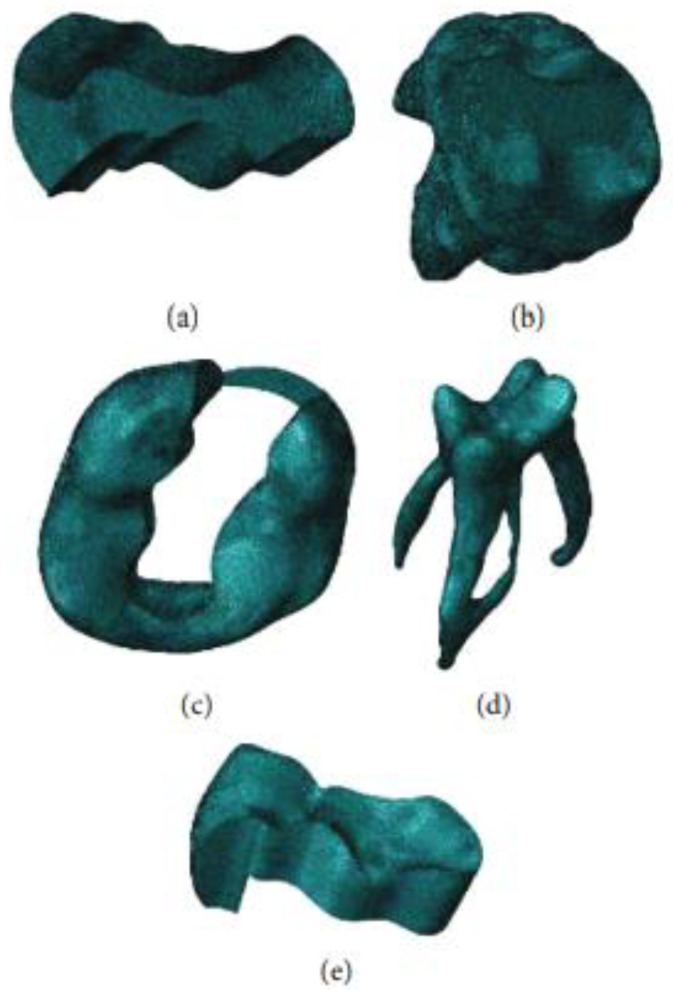
Geometric construction of molar tooth, restoration, and adhesive layer (class II disto-occlusal cavity: (**a**) adhesive layer, (**b**) dentine, (**c**) enamel, (**d**) pulp, (**e**) restoration).

The three-dimensional objects of each mask were converted to STL files after they were created. The obtained STL files were transferred to the Geomagic Design X program (Geomagic Design X 2020.0), necessary adjustments such as smoothing were made, and STP files were obtained. The obtained STP files were transferred to the Solidworks program (Solidworks corp., Waltham, MA, USA), cavity preparation was performed, an adhesive material layer was formed (Figure 3), and the obtained elements were transferred to the ABAQUS program (2020 Dassault Systems Simulation Corp., Johnston, RT, USA). The FE model was created in the ABAQUS program, and the stress distributions were analyzed.

**Figure 4:**
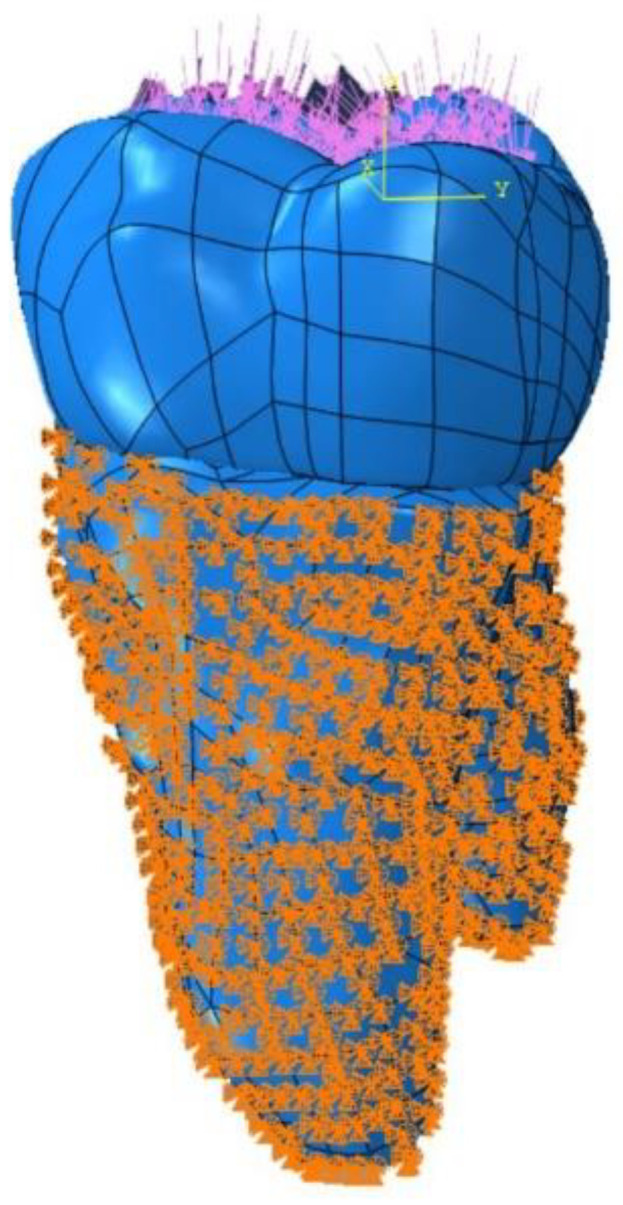
Load and boundary conditions.

A force of 600 N was applied to the restored tooth to compare the stress distributions in dental tissues and dental materials as a result of the applied occlusal forces (Figure 4). The selected value was within the physiological range of the occlusal forces on the molars [30].

As the periodontal ligament (PDL) was not modeled, fixed and pinned boundary conditions were utilized to simulate the roots that are fixed in the bone [31,32]. A single tooth and tooth type were used without simulating the periodontal ligament or bone. The mechanical boundary conditions (symmetry/antisymmetry/encostre) were selected using the “create boundary condition” tab in the load part of the Abaqus program. The effect of the periodontal ligament was ignored, and the tooth was pinned (U1 = U2 = U3 = 0) from the enamel–cementum junction to the apical region (Figure 4).

The amount of load applied to the tooth was selected according to the average fatigue values of restored molar teeth obtained from previous in vitro studies. The criteria for evaluating the fatigue life of restorative material, enamel, and dentin were compared to the maximum principal stress–life (S–N) diagram that occurs during loading. The S–N curve represents the strain amplitude ($\sigma_{0}$) as a function of the number of cycles to fracture (N).

Fatigue values for the filling materials were determined with three-point bending tests, as in previous studies. The fatigue behavior of the materials was calculated using the nonlinear Basquin formula, as shown in Equation (1) [31].

(1) $$\sigma_{a}=A{\left(N\right)}^{B}$$

The A and B values for the filling material are shown in Table 1. Wöhler curves for enamel and dentin were taken from the literature. These were drawn ($\sigma_{m}=0$) purely for reverse loading. Any other average voltages ($\sigma_{m}\neq 0$) are mathematically represented in Equation (2).

(2) $$\sigma_{a}=(\sigma_{f}-\sigma_{m}){\left(2N\right)}^{b}$$

The fixed values of $\sigma_{f}$ and b for dentin and enamel are shown in Table 1. Parts of FEA models need adequate information, especially regarding human oral system material properties. However, it is still difficult to determine the appropriate biomechanical environments of living tissues, especially dental health, in FEA. As well as confirming these observations, the wide range of values in the literature for the elastic modulus also proves these data. There are many factors that indicate the examination of the mechanical properties of restoration materials, including teeth. It has been seen in previous studies that the mechanical properties between materials of the same class vary greatly. The mechanical properties of dental tissues and restorative materials are shown in Table 2.

The mesh, nodes, and elements used in the FEA for the tooth, restoration, and adhesive layer are presented in Table 3. The analysis was initiated after the geometry and appearance of the mesh were properly meshed and regularized. To achieve the desired number of elements, the main parameter, which was the maximum principal stress, was taken into account. In the subsequent step, the number of elements was doubled, and the effect of this mesh reduction on the mentioned parameter was investigated. This process was repeated until a compromise between time and resources was achieved, without any significant changes in responses with the increase in the number of new networks. At this stage, it was concluded that the solutions converged and that there was no need to use more elements. Increasing the number of elements did not help to enhance the accuracy of the solution, but only prolonged the solution process.

## 3. Results

When the bulk–fill composite material was used in a class II disto-occlusal cavity, enamel was exposed to the most stress as a result of the occlusal forces on the tooth, followed by dentin and restorative material. The adhesive material received the least amount of stress. When the conventional resin composite material was used in the same cavity, the highest level of stress occurred on the enamel, followed by the dentin and restorative material, while the adhesive material underwent the least stress.

**Figure 5:**
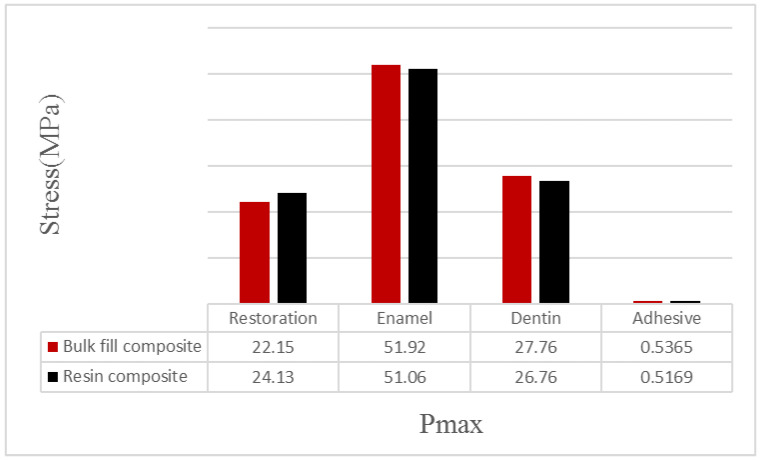
Stress distributions in tooth components restored as a result of occlusal forces.

**Figure 6:**
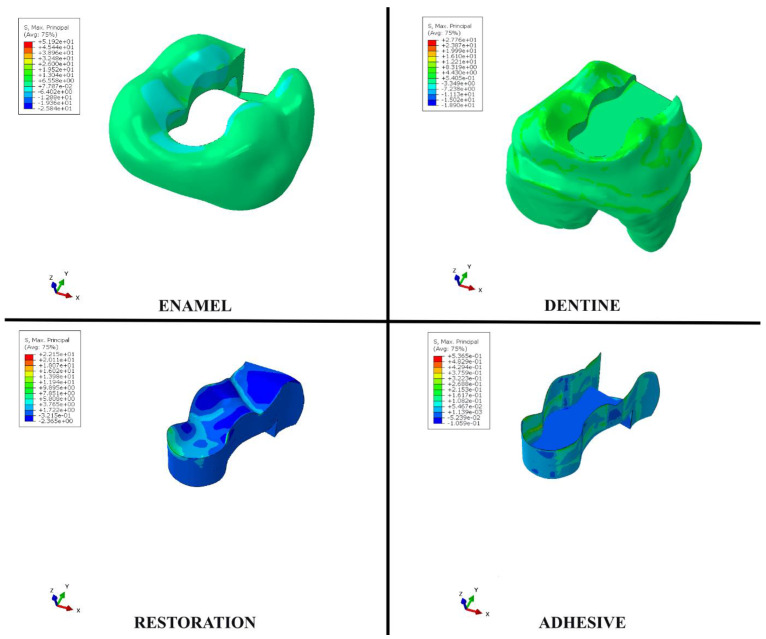
Distribution of stress areas in enamel, dentin, restoration and adhesive material when bulk−fill composite is used as a restorative material.

**Figure 7:**
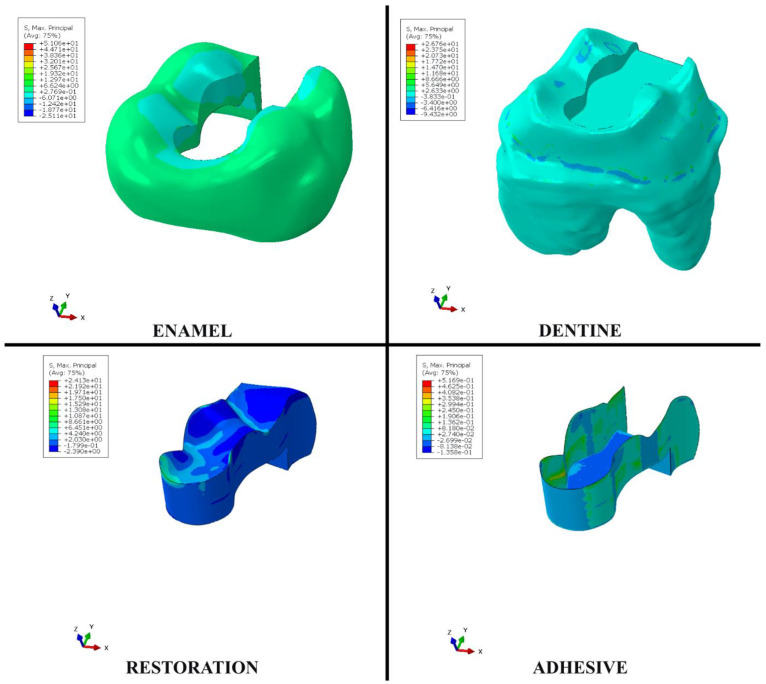
Distribution of stress areas in enamel, dentin, restoration, and adhesive material when composite resin is used as a restorative material.

When the bulk–fill composite material was used instead of the conventional resin composite material in the disto-occlusal cavity, the stress values on enamel, dentin, and adhesive materials increased as a result of occlusal forces. However, the amount of stress on the restorative material decreased. When the bulk–fill composite material was used instead of the conventional resin composite material in the cavity, the stress value increased from 51.06 to 51.92 in the enamel tissue, from 26.76 to 27.76 in the dentin tissue, and from 0.5169 to 0.5365 in the adhesive material. In addition, the amount of stress on the restorative material decreased from 24.13 to 22.15 (Table 4, Figure 5). In other words, when the bulk–fill composite material was used instead of conventional resin composite, it had a more destructive effect on enamel tissue, dentin tissue, and adhesive material; in contrast, there were less destructive effects on the restorative material because less stress was placed on it. It was concluded that when both the bulk–fill composite and conventional resin composite materials were used, the areas of low stress on the enamel were on the occlusal surface in the adjacent areas close to the restoration and on the tubercle ridges. In restorations in teeth with disto-occlusal class II cavities, the areas under the most stress were on the restoration surfaces adjacent to the enamel on the mesial marginal ridge and in the central fossa areas. In the adhesive material, the areas under the most stress were the parts located on the side walls of the adhesive adjacent, while there were lower levels of stress on the bottom of the adhesive compared to the amount of stress on the side walls (Figure 6 and Figure 7).

As a result of the forces that occur in the mouth, dental tissues and restorative materials are exposed to many harmful stresses. As a result of the continuity of these stresses, fractures occur in dental tissues and restorations. When the bulk–fill composite material was used in a class 2 disto-occlusal cavity, the number of cycles required for the restoration material to break as a result of the forces acting on the tooth–restoration complex was 1.583 × 10^32^. This was much more than the number of cycles required to break enamel and dentin. When the bulk–fill composite material was used, the number of cycles required to break the dentin tissue was 7.252 × 10^8^, and the number of cycles required to break the enamel tissue was 45.047 × 10^6^. In other words, while the restorative material was shown to have the longest fracture life as a result of the stresses caused by occlusal forces in the oral environment, enamel tissue had the shortest fracture life.

When the conventional resin composite material was used in a class II disto-occlusal cavity, the number of cycles required for the restoration material to break as a result of the forces acting on the tooth–restoration complex was 8.067 × 10^34^. This was much more than the number of cycles required to break the enamel and dentin tissue when the bulk–fill composite material was used. When the composite resin was used, the number of cycles required to break the dentin tissue was 6.103 × 10^9^, and the number of cycles required to break the enamel tissue was 54.369 × 10^6^. Based on these values, it can be seen that the fracture life of the restorative material is much longer than the enamel and dentin tissue as a result of the forces acting on the tooth tissues and restorative material. Enamel tissue has the shortest fracture life compared to the other materials.

When the bulk–fill composite material was used instead of the conventional resin composite material in the disto-occlusal cavity, the number of cycles required for enamel fracture decreased from 54.369 × 10^6^ to 45.047 × 10^6^ as a result of the forces generated in the oral environment. The number of cycles required for dentin fracture decreased from 6.103 × 10^9^ to 7.252 × 10^8^, and the number of cycles required to break the restorative material decreased from 8.067 × 10^34^ to 1.583 × 10^32^ (Table 5).

## 4. Discussion

In our study, the null hypothesis was rejected, as it was concluded that restorative materials can change the amount of stress on dental tissues and affect the fracture life of dental tissues.

In dentistry today, many restorative materials have been produced to make healthy dental tissues strong and resistant and to maintain their durability. Knowledge of the biomechanics occurring between teeth and in the mouth and making restorations by paying attention to this increases the lifespan and success of restorations. One of the most important factors in the success of the treatments in dentistry is the amount of force imposed on dental tissues [44]. The teeth in the posterior region are exposed to functional and parafunctional forces of different sizes and directions [45]. The forces generated in the mouth are very variable and have been reported to range from 10 N to 431 N [46]. At the same time, many researchers have clearly shown that oblique forces generate more stress than forces directed along the long axis of the tooth [47,48,49]. Fractures in restorative materials can sometimes be caused by interatomic interactions due to errors that occur during polymerization. In a study conducted in 2015, by Tian et al. examined the atomic movements of glass ionomer cements during curing and showed that these movements could lead to breakages in materials [50]. The development of computer technology and modeling techniques has made the finite element stress analysis method very reliable and important in experimental applications. The finite element stress analysis method has started to be increasingly used in the evaluation of force analysis, especially in cases where an accurate and fast response cannot be obtained from experimental methods [21,22]. The three-dimensional imaging of tooth structures in finite element stress analysis is performed using a limited number of points. The stresses, compressions, and strains are firstly separately calculated for the element body at each of these points and then as a whole, and conclusions are drawn [34].

Most conventional composite resins present similar elastic moduli to dentin; adhesive restorations can help to compensate for stress generated by dental tissue loss. Although widely used, composite restorations present some limitations, such as polymerization shrinkage, marginal discoloration, microleakage, and postoperative sensitivity [51,52,53].

All of the layering techniques show the effects of polymerization shrinkage, but many studies have shown that the incremental layering technique results in better performance than bulk placement in terms of polymerization shrinkage [54,55,56,57]. 

Ausiello et al. showed that tubercle displacement due to stress caused by polymerization shrinkage was greater when rigid composites were used in class II restorations than when more flexible composites were used [58]. The bond strength of the adhesive is a very important factor that influences the overall mechanical properties of a restoration by improving interlayer bonding, debonding resistance, and fatigue resistance [59]. 

In this study, the stress distribution and fracture lifetimes of resin-containing composite and bulk–fill composite materials, which are frequently used in dentistry, in disto-occlusal class II cavities in first molar teeth, were compared with the three-dimensional finite element stress analysis method.

Dynamic properties, such as the fatigue resistance of resin composites, were investigated by McCabe et al. For resin composites, a significant decrease in mechanical strength occurred after 1 × 10^4^ cyclic loading [60]. In another study conducted in 1997, dynamic properties, such as the fatigue resistance of resin composites and compomers, were investigated. They found a significant decrease in mechanical strength occurred in resin composite and compomer materials after 1 × 10^5^ cyclic loading [61]. 

The results of the study by Kuijs et al. suggest that ceramic, indirect resin composite and direct resin composite restorations provide comparable fatigue resistance and exhibit comparable failure modes in the case of fracture, although indirect restorations tend to fracture more cohesively than direct restorations [62]. 

de Kok et al. concluded that composite resin exhibits a significantly higher level of fatigue resistance than glass ceramic when bonded to dentin. On enamel, no significant difference was found between the two materials. In other words, thin direct composite resin restorations on dentin were more durable than glass ceramics [63]. 

Yamanel et al. evaluated the effects of restorative materials and cavity designs on tooth structures and stress distribution in restorative materials through three-dimensional finite element analysis. They created three-dimensional inlay and onlay cavity designs in first molar teeth and used two different composites with nanofil filler and two different all-ceramic materials. As a result of the study, it was shown that materials with low elastic moduli transferred more stress to tooth structures; that is, compared to composites with nano-fillers, the tested all-ceramic inlay and onlay materials transferred less stress to tooth structures. In addition, the onlay design was more effective in protecting the tooth structures than the inlay design [20]. Pest et al. stated that more rigid restorative materials are more resistant to stress, but transfer most of the functional stress to the less rigid dentin, thus increasing the risk of root fracture [64]. Mesquita et al. reported that a composite with a low elastic modulus will be more deformed in the face of functional stresses, and as a result, the fracture of the tooth structure and the weakening of the connection between the tooth and the restoration will lead to postoperative sensitivity and secondary caries [17]. The filler ratio in restorative materials also affects the fatigue and fracture of the material. Htang et al. described a correlation of filler content in restorative material on fatigue strength. However, with a filler fraction of 75% by weight, the maximum fatigue resistance was determined [65]. Collins et al. evaluated the clinical survival of posterior resin composite restorations. As a result of the analysis of clinical data, they found that microfills may be more susceptible to bulk breakage [66]. Shembish et al. investigated fatigue resistance in resin composite CAD/CAM crowns. The crowns showed only minor occlusal damage during gradual stress loading up to 1700 N. The damage that occurred was limited to the restoration and did not cause any damage to the tooth structure. The areas with the highest failure rates were similar regions to those in our study [67]. 

Batalha-Silva et al. investigated the stress distribution in vitro after applying composite restorations to molar teeth with MOD cavities under loads of 200 N (5000 cycles) followed by 400, 600, 800, 1000, 1200, and 1400 N (30,000 cycles each; 185,000 cycles in total). Teeth restored with composites fractured under an average load of 1213 N. Only two of the 50 samples prepared withstood all the incoming loads. In these samples, the failures were generally observed in the enamel–cementum junction areas [68]. 

In 2023, Thaungwilai et al. compared the stresses on the periodontal ligament, dentin, and stainless-steel crown with finite element stress analysis after covering the composite core-applied primary molars with a stainless-steel crown. In the study, the most stressed areas in the occlusal region were the parts of the central fossa, as shown in our study [69]. 

The stress distribution values in the resin composite and bulk–fill restorations in our study are shown in Figure 6 and Figure 7. When we compared the results of finite element stress analysis with the in vitro studies of Magne et al. in 2010 and Garcia-Godoy et al. in 2012, good agreement was observed between areas of maximum stress and unsuccessful regions. Common failures of restorations occurred in enamel contact areas or parts of the central fossa [70,71].

## 5. Conclusions

In our study, we investigated how the materials to be used in restorations affect the amount of stress and the fracture lives of enamel, dentin, and restorative materials, and we reached two important conclusions as a result of the study.

1—When the bulk–fill composite material was used instead of the conventional resin composite material in the disto-occlusal cavity, the stress values on the enamel, dentin, and adhesive material increased as a result of occlusal forces, while the amount of stress on the restorative material decreased. In other words, when the bulk–fill composite material was used instead of the conventional resin composite material, there were more destructive effects on enamel tissue, dentin tissue, and adhesive material, while the restorative material was exposed to less destructive effects.

2—In the fracture analysis, when the bulk–fill composite material was used instead of the conventional resin composite material, a decrease in the number of cycles required for the fracture of the enamel, dentin, and restorative materials was observed as a result of the forces generated in the oral environment. In other words, the use of the conventional resin composite material instead of the bulk–fill composite material increased the fracture lives of enamel tissue, dentin tissue, and restorative material against forces.

## Figures and Tables

**Table 1:** Coefficient and exponent constants of fatigue curves of dental materials and tooth tissues [30,33,34,35,36].

Material	A (MPa)	B	$\sigma_{f}\;(\mathrm{MPa})$	b
Enamel			310	−0.111
Dentin			247	−0.111
Bulk–fill composite	54	−0.020		
Resin composite	84	−0.035		

**Table 2:** Mechanical properties of dental tissues and restorative materials [37,38,39,40,41,42,43].

Material	Young’s Modulus (GPa)	Poisson’s Ratio	Compressive Strength (MPa)	Flexural Strength (MPa)	Shear Strength (MPa)	Fracture Toughness (Mpa m^1/2^)	Microhardness (H_V_)
Enamel	84.1	0.33	384	11.5	60	0.8	3–6
Dentin	18.6	0.31	297	105.5	12–138	3.08	0.13–0.51
Adhesive	1	0,24					
Pulp	0.002	0.45					
Bulk–fill composite	12	0.25	169	42			
Resin composite	16.6	0.24	294	77			

**Table 3:** Nodes and elements for tested groups.

Total Elements	Total Nodes	Mesh Type
7,428,602	1,368,958	Linear tetrahedral elements of C3D4

**Table 4:** Stress distributions in tooth components restored as a result of occlusal forces.

Restoration Material	Restoration	Enamel	Dentin	Adhesive
Bulk–fill composite	22.15	51.92	27.76	0.5365
Resin composite	24.13	51.06	26.76	0.5169

**Table 5:** Estimated number of cycles required for fracture of dental materials and dental tissues as a result of forces.

Material Group	Restoration	Enamel	Dentin
Bulk–fill composite	1.583 × 10^32^	45.047 × 10^6^	7.252 × 10^8^
Resin composite	8.067 × 10^34^	54.369 × 10^6^	6.103 × 10^9^

## Data Availability

The data presented in this study are available upon request from the authors.
